# IL-6 Serum Levels in COVID-19 Patients With Vertigo

**DOI:** 10.7759/cureus.35042

**Published:** 2023-02-16

**Authors:** Dimitrios Kitsos, John Tzartos, George Korres, Vasileios Giannopapas, Maria Riga, Christos Stergiou, Anthi Tsoga, Christos Grigoropoulos, Georgios Paraskevas, Christina Zompola, Thomas Nikolopoulos, Sotirios Giannopoulos

**Affiliations:** 1 Department of Neurology, Attikon University Hospital, Athens, GRC; 2 Department of Otolaryngology, National and Kapodistrian University of Athens School of Medicine, Athens, GRC; 3 Physical Therapy, University of West Attica, Athens, GRC; 4 Department of Otorhinolaryngology-Head and Neck Surgery, Dammam Medical Complex, Dammam, SAU; 5 Department of Otorhinolaryngology, Attikon University Hospital, Athens, GRC; 6 Department of Otorhinolaryngology, Attikon University Hospital, National and Kapodistrian University of Athens School of Medicine, Athens, GRC

**Keywords:** interleukin (il)-6, long covid syndrome, dizziness, vertigo, covid-19

## Abstract

Introduction

Dizziness and vertigo represent well-established symptoms of COVID-19. An overexpression of cytokines, a condition often described with the term “cytokine storm” or “hypercytokinemia”, is a key characteristic of SARS-Cov-2 infection and plays a pivotal role in disease progression and prognosis. Among them, IL-6 is of major importance.

Purpose

The purpose of this study is to investigate any probable IL-6 serum titer difference in COVID-19 patients with vertigo (V+) or without vertigo (V-) admitted to the COVID-19 internal medicine departments of Attikon University Hospital, Athens, Greece, within 12 months.

Methods

The sample consisted of 52 COVID-19 patients who were diagnosed between January 1, 2020, and December 31, 2020. Of those, 31 reported vertigos during their admission (V+), while the remaining 21 COVID-19 patients did not complain of such symptoms (V-).

Results

Higher IL-6 serum levels post-COVID-19 infections lead to higher incidence rates of vertigo symptoms (p<.005), regardless of gender and age (p.005).

## Introduction

COVID-19 can range from asymptomatic or mild infection, resembling a common cold, to severe respiratory distress syndrome and fatal pulmonary failure. Dizziness represents a well-established group of symptoms that are encountered quite often in COVID-19 infection. Ranging from disequilibrium, presyncope, and lightheadedness to vertigo, 8% to 30% of COVID-19 patients report one or more of the above-mentioned symptoms, which are usually recorded with the term dizziness [1,2]. Even though the cause remains unknown, some of the hypothesized mechanisms by which SARS-CoV-2 may cause dizziness or vertigo include: dysautonomia (postural orthostatic tachycardia syndrome) [3], vestibular neuritis or labyrinthitis, microvascular injury, endothelial dysfunction, and decalcification-induced benign peripheral positional vertigo (BPPV) [4,5]. True vertigo, defined as the illusion of motion when there is none, can be either peripheral or central in etiology. The role of oxidative stress and inflammation in vestibular disorders has been extensively investigated. Studies have shown that the levels of IL-6 and TNF-α were significantly higher in patients with BPPV [6] and vascular vertigo when compared to controls and patients with vertigo of nonvascular etiology [7]. Cytokines are cell signaling proteins produced by a variety of immune cells, including macrophages, B and T lymphocytes, mast cells, dendritic cells, fibroblasts, and endothelial cells. They act through cell surface receptors, modulate the humoral and cell-mediated immune response, and can have pro- or anti-inflammatory properties [6]. Numerous studies have described abnormal levels of various cytokines in COVID-19 patients, including IL-1, IL-2, IL-4, IL-6, IL-7, IL-10, IL-12, IL-13, IL-17, M-CSF, G-CSF, GM-CSF, IP-10, IFN-γ, MCP-1, MIP 1-α, hepatocyte growth factor (HGF), TNF-α, and vascular endothelial growth factor (VEGF) [8-10]. Among them, IL-6 is of major importance. In those studies, the major player highlighted is IL-6, which represents an important mediator of the acute inflammatory phase response and has been used as a marker of COVID-19 severity [11]. 

The purpose of this study is to explore any possible relationships between IL-6 serum titers in post-COVID-19 patients with or without vertigo during the active infection phase who were admitted to the COVID-19 internal medicine departments of Attikon University Hospital, Athens, Greece, within a time frame of 12 months.

## Materials and methods

Consecutive post-COVID-19 patients were examined at the outpatient clinics of the Department of Neurology at Attikon University Hospital, and were asked to participate in the study and sign the corresponding declaration form. They were all admitted within the last 12 months at the COVID-19 departments of the Attikon University Hospital, and their appointment was scheduled within 15 to 30 days post-discharge and declaration of Sars-Cov-2 DNA PCR seronegativity. The study exclusion criteria were a) DNA-PCR seropositivity, b) COVID-19 central nervous system (CNS) infection, and c) internal care unit (ICU) admission due to Sars-Cov-2 complications.

The sample consisted of 52 COVID-19 patients who were diagnosed between the 1st of January 2020 and the 1st of December of the same year. Of those, 31 reported vertigo during their admission (V+), while the remaining 21 COVID-19 patients did not complain of such symptoms (V-).

During a study appointment, a complete neurological exam was performed, and 25 ml of blood was collected and centrifuged within an hour. All specimens were spun at the same time at 3500 pg for 15 minutes. The samples were stored at 1ml aliquots at -70 degrees Celsius until the performance of the IL-6 immunoassay. IL-6 immunoassays were performed on the Lumipulse G600II using the chemiluminescence enzyme immunoassay technology for the quantitative determination of IL-6. The study was performed according to the Helsinki Declaration and was approved by the Institutional Review Board of the University General Hospital Attikon. All patients signed informed written consents during the appointment.

## Results

The sample consisted of 52 post-COVID-19 patients (51.9% female, 41.8% male) with a mean age of 34.2 (min=19.0, max=34.2, std=11.6) and a mean IL-6 of 1.40 in the V- group and 1.93 in the V+ group (Table 1). An independent samples t-test was performed between the two groups of V+ and V- patients and IL-6 levels. The test showed a difference in the mean IL-6 levels of the two groups (p=.039) (Table 2). Sequentially, a binary logistic regression was performed in order to identify any possible correlations between IL-6 levels and post-COVID-19 vertigo status. The model passed the Omnibus test (χ2(1,N=52)=4.35, p=.037), and it appeared to fit the data set according to the results of the Hosmer & Lemeshow test (p=.41). The model had medium accuracy (65.4) and explained 10.8% (Nagelkerke R2) of the variance of post-COVID-19 vertigo, and based on the normalized residual table frequencies, we identified one outlier in the dataset with an absolute value of 2.2. According to the results of the binary logistic regression, IL-6 was identified as the only significant predictor with an adjusted OR of 1.9 and a CI [1.004, 3.89] (p=.049), indicating that patients with higher IL-6 serum levels were twice as likely to be V+ (Table 3). The variables age and gender did not appear to affect the odds of post-COVID-19 vertigo. 

**Table 1:** Demographic Characteristics

	M/F	SD/Percentage
Gender		
Female	27	51,9%
Male	25	48,1%
Age	34,2	11,62
IL-6 serum levels		
V+	19.3	0.90
V-	1.40	0.86

**Table 2:** Independent Sample T-Test

		Levene’s				t-test			
IL_6 -Vertigo	F	Sig	t	df	Sig. (1)	Sig (2)	MD	SE	95% CI
	.589	.447	-2.22	50	.019	.039	-.528	-.249	[-1.02,-0.2]

**Table 3:** Adjusted Odds Ratios

	B	s.e	Wald	Df	Sig	Exp(B)	95% CI Exp(B)
IL-6	.681	.346	3.883	1	.049	1.97	[1.004-3891]
Constant	-1.512	.642	5.546	1	.019	.220	

## Discussion

The results of this study reached a significant conclusion where higher IL-6 serum level post-COVID-19 infection led to a higher incidence of vertigo symptomatology, accounting for 10.8% of the variance (V+ status). More specifically, patients with higher IL-6 serum levels had a two-fold increase in the manifestation of post-COVID-19 vertigo (V+). Participants’ age and gender did not appear to affect the manifestation of vertigo symptomatology.

An overexpression of cytokines, a condition often described with the term cytokine-storm or hypercytokinemia, is a key characteristic of SARS-Cov-2 infection and plays a pivotal role in disease progression and prognosis [9,10]. Elevated levels of proinflammatory effector cytokines, such as tumor necrosis factor (TNF), IL 1-β, IL-6, and IL-8, have been reported in patients with COVID-19 disease [12], indicating a link of the viral spike particle towards the ACE 2 receptor [13] which may result in blood-brain barrier (BBB) impairment and consequently neurological deficits [14]. In addition, proposed pathophysiologic mechanisms of COVID-19-induced vertigo include a) direct viral infection or inflammation of the inner ear and cochleovestibular nerve causing vestibular neuritis, labyrinthitis, or BPPV, b) vascular compromise due to microthrombi formation or vasculitis and endothelitis and c) the vestibulotoxic effect of medications used in the treatment of COVID-19.

The proposed pathophysiologic mechanisms are based on the work on the effect of cytokines in BPPV. The levels of IL-1β, IL-6, and TNF-α were higher in BPPV patient groups and decreased with the repositioning maneuver. The decreases in the IL-1β and IL-6 levels were statistically significant after treatment. Like oxidative stress, the primary elevation of inflammatory mediators may cause otolith formation or otolith migration to the semicircular canals, or the vertigo attack itself in BPPV may cause an increase in inflammatory mediators [6]

Neurological involvement manifestation, such as vertigo or dizziness, has been reported as a clinical sign and potential consequence of COVID-19. With a growing population of recovering patients, it was evident that in a certain amount of patients (up to 32%), vertigo and or dizziness may persist beyond the acute phase of infection [15,16]. The pathophysiology of vertigo manifestation as a post-acute COVID-19 infection symptom following a mild or moderate infection is still largely unclear. While it may be a consequence of virus-induced tissue injury, another potential trigger has been proposed to result from persistent SARS-CoV-2 reservoirs [15]. To make things even more complex, it seems that vertigo symptoms related to connective tissue autoimmune disease (systemic lupus erythematosus, rheumatoid arthritis) seem to exacerbate post-COVID-19 infection and during the long COVID period via the production of specific autoantibodies [15].

Consequently, dizziness may appear in the other phases of the infection (i.e., post-infection manifestation) via alternate pathophysiological mechanisms). In that multi-faceted pathway which leads to vertigo via autoimmunity, exacerbation of vertigo due to autoantibodies in the case of autoimmune diseases creates an even more versatile environment where several reversible (cycle of the Sars-Cov2 virus, administration of anti-COVID-19 antivirals, vaccination status) and non-reversible (history of autoimmune disease, type of autoimmunity and demographic factors) factors seem to contribute to the manifestation of a similar from the clinical practitioner point of view but totally different in its clinical characteristics symptom of vertigo from the patient point of view (severity, answer to medication, duration and fluctuation of the symptom) [17].

In a recent study, the presence and severity of tinnitus and vertigo were assessed in a large cohort of long COVID-19 patients. Neurological symptoms were prevalent, with 60% of long COVID reporting some degree of vertigo or dizziness. The need for collaborative treatment techniques in long COVID-19 clinics, including experts in otorhinolaryngology, is highlighted by the high prevalence of neurotological symptoms such as vertigo [18].

Recent research has highlighted the significance of neuroinflammation in “long-COVID,” indicating that abnormal humoral and cellular immune responses, interleukin-6 (IL-6), and other systemic inflammatory markers, as well as autoantibodies that target cellular receptors, may play a role in systemic and neurological "long-COVID" sequelae [19].

Regarding the limitations of the study, the sample size was quite small, hence the correlation cannot be established with certainty. There was no heterogeneity regarding age and vertigo history, and there has been no reference to the gravity of the symptom, the diagnostic work, and any treatments given (pharmaceutical or not). Finally, the follow-up of the symptoms has not yet reached a period longer than six months and was not within the same time frame for all participants.

## Conclusions

To the authors’ knowledge, there are no similar studies investigating a correlation between interleukin levels and vertigo during COVID-19 infection. Further follow-up, which will not be tackled by the limitations discussed above, is advised regarding vestibular testing and monitoring of the IL-6 levels since vertigo is also considered one of the most frequent long COVID-19 symptoms, and the status of the pandemic at the current time allows more systematic diagnostic work up and investigation of such symptoms. A larger sample of patients with a history of vertigo should be monitored following COVID-19 in order to verify our findings.
